# When Does Work Interfere With Teachers’ Private Life? An Application of the Job Demands-Resources Model

**DOI:** 10.3389/fpsyg.2019.01121

**Published:** 2019-05-21

**Authors:** Alessandro De Carlo, Damiano Girardi, Alessandra Falco, Laura Dal Corso, Annamaria Di Sipio

**Affiliations:** ^1^Giustino Fortunato University, Benevento, Italy; ^2^FISPPA Section of Applied Psychology, University of Padova, Padua, Italy

**Keywords:** work-family conflict, teachers, workload, support, participation, moderation

## Abstract

The purpose of this study is to examine the relationship between contextual work-related factors on the one hand, in terms of job demands (i.e., risk factors) and job resources (i.e., protective factors), and work-family conflict (WFC) in teachers on the other. Building on the Job Demands-Resources (JD-R) model, we hypothesized that job demands, namely qualitative, and quantitative workload, are positively associated with WFC in teachers. Moreover, in line with the buffer hypothesis of the JD-R, we expected job resources, in terms of support from supervisor (SS), job autonomy (JA), and participation in decision making (PDM), to affect this association, which is expected to be stronger when job resources are low. The study was conducted in an Italian secondary school. Overall, 122 teachers completed a self-report questionnaire aimed at determining WFC, as well as job demands and resources. The hypothesized relationships were tested using moderated multiple regression. The results of this study largely support our predictions. First, both aspects of workload were positively associated with WFC. Secondly, job resources, including SS and PDM, buffered this association, which was stronger when resources were low. On the contrary, JA did not buffer the association between workload and WFC. Overall, the results of this study are consistent with the JD-R model and contribute to the understanding of work–family conflict among teachers. More specifically, our study suggests that teachers with high levels of job resources, namely SS and PDM, can effectively cope with job demands, in terms of both qualitative and quantitative workload, thus preventing negative consequences such as conflict between work and family domains. Interventions aimed at preventing WFC among teachers should encourage organizations to optimize the balance between job demands and resources, as well as the identification and training of the workers at risk of WFC.

## Introduction

Over the past decades, the teaching profession has undergone several changes, often described in the literature as intensification (Hargreaves, 1992; Ballet and Kelchtermans, 2009; Van Droogenbroeck et al., 2014). According to this perspective, teachers are increasingly exposed to external expectations and pressures (e.g., from supervisors, parents, and policy makers), which result in higher workload, both teaching- and non-teaching-related (e.g., administrative work), and less time for interactions with colleagues as well as for one’s private life. This may lead to a chronic feeling of work overload, both at school and at home, to the loss of specific professional skills, and to work-related stress.

Not surprisingly, work-family conflict (WFC) seems to play a central role in the stress process among teachers. Indeed, several job characteristics, such as workload, job insecurity, emotional labor, emotional investment in students’ behavioral problems, and demanding interactions with parents, are associated with WFC among teachers (Cinamon et al., 2007; Noor and Zainuddin, 2011; Ilies et al., 2015; Richter et al., 2015; see also Michel et al., 2011, for a meta-analysis across different occupations). WFC may in turn have negative consequences for both teachers and their students, in terms of teachers’ job burnout, poorer psychological and physical health, reduced organizational citizenship behaviors and job satisfaction, as well as lower students’ perceived teacher autonomy support and autonomous motivation (Bragger et al., 2005; Cinamon and Rich, 2010; Bell et al., 2012; Guglielmi et al., 2012; Gao et al., 2013; Haslam et al., 2013; Santisi et al., 2014; Benevene and Fiorilli, 2015; Fiorilli et al., 2015; Shen et al., 2015; Bélanger et al., 2016; see also Amstad et al., 2011, for a meta-analysis across different occupations).

Therefore, in order to prevent illness and promote well-being and job performance among teachers, it seems important to investigate those precursors that may foster or prevent WFC. In this study, in light of the theoretical framework provided by the Job Demands-Resources model (JD-R; Demerouti et al., 2001; Bakker and Demerouti, 2007, 2017), we examined whether job demands (i.e., risk factors) are positively associated with WFC among teachers in an Italian secondary school, and whether job resources (i.e., protective factors) can moderate such association (Bakker et al., 2011). Previous research has shown that teachers in Italian secondary schools experience less work life-balance than those employed at other educational stages (Magnano et al., 2014b).

According to Greenhaus and Beutell (1985), WFC may be defined as a type of interrole conflict in which role pressures arising from work and family domains are mutually incompatible to some degree. This definition is consistent with the role stress theory (Kahn et al., 1964), according to which the resources available to individuals, such as time and energy, are limited, and the fulfillment of multiple roles unavoidably leads to the depletion of these resources. Therefore, individuals have to allocate their limited resources over work and family domains, in order to minimize the strain inexorably associated with the management of multiple roles (Geurts and Demerouti, 2003). Greenhaus and Beutell (1985) identified three dimensions of WFC, namely time-based, strain-based, and behavior-based conflict. According to this classification, the time devoted to one role, the strain produced by one role, or the behaviors required by one role, respectively, make it difficult to meet the demands of the other role. The interference between work and family domains may occur in both directions. In this regard, Netemeyer et al. (1996) proposed a distinction between WFC and family work conflict (FWC). WFC occurs when the demands of one’s job, the time dedicated to one’s job, and the strain generated by one’s job interfere with responsibilities related to the family. Conversely, FWC refers to the interference between family demands, time dedicated to family activities, and strain created by the family on the one hand, and work-related responsibilities on the other. In this study, we focused exclusively on WFC, because we are interested in the effects of work context, in terms of job demands and resources, on teachers’ functioning in the family domain.

**FIGURE 1:**
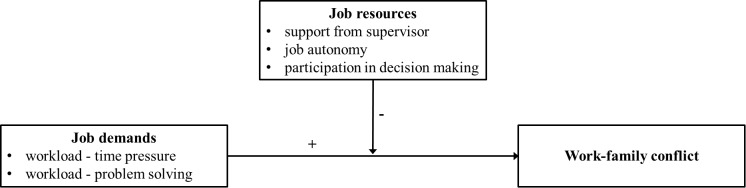
An application of the Job Demands–Resources model to work-family conflict (WFC) in teachers.

The JD-R (Demerouti et al., 2001; Bakker and Demerouti, 2007, 2017) is a flexible model of job stress and well-being (Schaufeli and Taris, 2014) that has recently been applied to the interface between work and private life (Bakker et al., 2011). According to the JD-R, job characteristics of different occupations may be classified either as job demands or job resources. On the one hand, job demands are those aspects of a job (physical, psychological, social, and organizational) that require sustained physical and/or psychological (cognitive and emotional) effort from the employee and are therefore associated with certain psychological and/or physiological costs. On the other hand, job resources are those aspects of a job (physical, psychological, social, and organizational) that are functional in achieving work goals, reducing job demands and the associated costs (psychological and/or physiological), or promoting personal growth, learning, and development (Demerouti et al., 2001; Bakker and Demerouti, 2007, 2017). In line with the JD-R, high job demands may increase WFC due to the depletion of personal resources (e.g., time, physical and emotional energy; Bakker et al., 2011). If an individual has few resources available at the end of the day due to energy depletion at work, he or she will be less likely to be involved in his or her family role at home, which may give rise to work–family conflict (Ilies et al., 2015). Furthermore, the buffer hypothesis of the JD-R claims that high job resources may offset the harmful impact of job demands on work family conflict (Bakker and Demerouti, 2007, 2017; Xanthopoulou et al., 2007). Accordingly, specific job designs in which teachers are burdened with high job demands, but lack adequate levels of job resources, are particularly likely to increase WFC (Bakker et al., 2011). The conceptual model is depicted in Figure 1.

That being said, there are different reasons why distinct job demands and job resources may influence WFC in teachers. More specifically, in this study two different facets of workload were considered as job demands, namely time pressure and problem solving. The former reflects quantitative workload, the amount of work to be done in a given time, whereas the latter refers to qualitative workload, which pertains to the difficulty or complexity of the job, for which the worker is not trained or does not have enough resources to deal with (Xie et al., 2008; Bowling and Kirkendall, 2012). We focused on these specific job demands because previous research has shown an association between said factors and WFC (Byron, 2005; Michel et al., 2011). Moreover, in line with the definition proposed by Greenhaus and Beutell (1985), we expect quantitative and qualitative workload to influence two relevant dimensions of the construct, namely the time- and strain-based ones (Geurts and Demerouti, 2003; van Hooff et al., 2005). Finally, these two facets of workload reflect important job demands for teachers that are acknowledged in the literature, such as time pressure, heavy workload (e.g., the necessity to prepare for teaching in the evenings or weekends), the diversity of tasks required (e.g., teaching- and non-teaching-related workload), and bureaucracy (Mearns and Cain, 2003; Skaalvik and Skaalvik, 2010; Antoniou et al., 2013; Van Droogenbroeck et al., 2014).

With respect to job resources, in this study we focused on support from supervisor (SS), job autonomy (JA), and participation in decision making (PDM) for several reasons. First, at a more general level and in line with the Self-Determination Theory (Deci and Ryan, 2000; Deci et al., 2017), these job resources may promote the satisfaction of basic human needs, such as, respectively, the need of relatedness (i.e., feeling part of a group, loved, and cared for), autonomy (i.e., to experience a sense of psychological freedom and volition), and competence (i.e., feeling effective in the interaction with the environment; Van den Broeck et al., 2010). The satisfaction of these needs, in turn, may lead to motivation and work-engagement (Van den Broeck et al., 2008), which help workers to cope effectively with job demands, thus preventing negative consequences such as WFC and job burnout (Xanthopoulou et al., 2007; Bakker et al., 2011).

Furthermore, there are other specific reasons why these job resources may buffer the relationship between job demands, in terms of qualitative and quantitative workload, and WFC in teachers. JA is associated with more opportunities to cope with job demands, and it might be of crucial importance for teachers, who can develop their own strategies to deal with educational (e.g., selecting student goals and their own teaching methods), and administrative demands (e.g., paperwork, meetings, and accountability demands; Van Droogenbroeck et al., 2014; De Neve et al., 2015). With respect to SS, school leaders such as principals and vice principals play a central role in supporting teachers, providing them with emotional and instrumental support (e.g., encouraging teachers to collaborate, providing support for policy changes and during conflicts with students or parents, providing extra job resources or opportunity for professional development; Kossek et al., 2011; Honingh and Hooge, 2014; Bélanger et al., 2015). Moreover, several facets of organizational support are associated with higher job satisfaction and reduced psychological strain, which may help to prevent negative consequences for the individual such as WFC (Riggle et al., 2009; Cortini et al., 2016). Finally, encouraging PDM may help teachers to affect the organization of their work, providing them with the opportunity to influence the allocation of resources, to deal with external or internal pressures, or to reduce hindrances to job performance, therefore increasing their effectiveness, both in the educational and the school administration domain (Bakker et al., 2007; Sarafidou and Chatziioannidis, 2013). This is particularly relevant in shifting and unstable work environments, in which the collaboration of different players is useful to create more effective work practices (Scaratti et al., 2017).

Therefore, based on these arguments and given the assumptions of the JD-R, we hypothesized that job demands will be positively associated with WFC, and that job resources will affect this association, which will be particularly strong when JR are low.

Hypothesis 1: problem solving (H1a) and time pressure (H1b), two facets of workload that reflect job demands, will be positively associated with WFC;
Hypothesis 2: the association between problem solving and WFC (H2a), as well as the association between time pressure and WFC (H2b), will be moderated by job resources, in terms of SS, JA and PDM, so that job resources will attenuate the positive association between job demands and WFC.

## Materials and Methods

### Participants and Procedure

The present study was conducted in an Italian secondary school as part of a work-related stress risk assessment. Participants were teachers, who were informed beforehand about the aims of the investigation and took part in the study on a voluntary basis. All participants gave their written informed consent before the administration of the questionnaire, in accordance with the Declaration of Helsinki. The study was carried out in accordance to the rules of AIP (Associazione Italiana di Psicologia – Italian Association of Psychology), according to which there was no need for previous ethics approval, since it would not deal with animals or vulnerable groups, or would involve risk for the well-being of participants, or use biomedical devices or invasive investigation tools. Our study did not need ethics approval, according to our national regulations as well as to the Ethics Committee of the University of Padova. A self-report questionnaire aimed at determining job demands, job resources, and WFC was administered. Overall, 150 teachers completed the questionnaire. However, 28 participants had missing values in at least one of the variables considered in the study and were therefore excluded from subsequent analyses. Accordingly, the final sample comprised of 122 participants. Fifty-four participants were women and 67 men (one missing value). Most respondents were aged between 46 and 55 years (47.6%), 31.9% were younger than 46 years, and 20.5% were older than 55 years. With respect to work experience, the majority of them (52.5%) had been teaching at the school for 10 years or less (46.7% for more than 10 years; one missing value). Most participants were married (70.5%) and had a permanent contract (83.6%). Finally, with respect to parental status, 52.4% of participants had two or more children, 23.8% had one child and 23.8% had no children.

### Measures

To determine the dimensions under investigation, the following self-report measures were administered. All the scales were taken from the Q-Bo test, an instrument standardized for the Italian context (De Carlo et al., 2008). The six-point response scale ranged from 1 (*strongly disagree*) to 6 (*strongly agree*).

*Job demands*, in terms of workload, were determined using ten items, designed to detect problem solving and time pressure (i.e., qualitative and quantitative workload). Examples of items were “*My job requires me to constantly solve new problems*” (problem solving, five items) and “*I have to work very fast*” (time pressure, five items). Cronbach’s alpha was 0.85 for problem solving and 0.87 for time pressure.
*Job resources* were assessed using fifteen items, aimed at determining SS (five items; e.g., “*My supervisor values the work I do*”), JA (seven items; e.g., “*I can organize my work autonomously*”), and PDM (three items; e.g., “*Teachers are involved in making important decisions and the definition of work goals*”). Cronbach’s alpha was 0.86 for SS, 0.92 for JA, and 0.63 for PDM.
*Work-family conflict* was determined using five items (e.g., “*I devote too little time to my family because of my job*”). Cronbach’s alpha was 0.89.

### Data Analysis

The hypothesized relationships were tested using moderated multiple regressions analyses following the procedure outlined by Aiken and West (1991); see also Cohen et al., 2003). WFC was the dependent variable, whereas job demands (i.e., problem solving and time pressure) and job resources (i.e., SS, JA, and PDM) were the independent and the moderating variables, respectively. The scores of each job demand and job resource were centered, and then the cross-products of centered variables were computed.

Overall, six different models were estimated. In Model 1 (M1), the centered scores of problem solving (JD) and SS (JR), as well as the interaction term, were entered in the regression model. In Model 2 (M2) and Model 3 (M3) JA (M2) or PDM (M3) were the job resources, respectively, whereas problem solving was the job demand. Model 4 (M4), Model 5 (M5), and Model 6 (M6) were similar, except that time pressure was the job demand. These models (M1–M6) were also estimated omitting the respective interaction term, to assess the additional variance explained by each of them.

To interpret the nature of the moderating effect, significant interactions were presented graphically, following the procedure outlined by Aiken and West (1991). Finally, if a significant interaction was found, then a simple slope analysis was conducted, to determine whether job demands are associated with WFC at high (+1*SD*) and low (-1*SD*) levels of job resources (Aiken and West, 1991). The analyses were performed using R version 3.5.1 (R Core Team, 2018).

## Results

Descriptive statistics and correlations between study variables are reported in Table 1. Interestingly, both time pressure (*r*_120_ = 0.51, *p* < 0.001) and problem solving (*r*_120_ = 0.40, *p* < 0.001), the two facets of workload that reflect job demands, were positively associated with WFC. Conversely, there was a negative association between WFC and job resources, in terms of SS (*r*_120_ = -0.36, *p* < 0.001), JA (*r*_120_ = -0.34, *p* < 0.001), and PDM (*r*_120_ = -0.25, *p* < 0.01).

**Table 1:** Means, Standard Deviations, and Correlations Between Study Variables (*N* = 122).

	*M*	*SD*	1	2	3	4	5	6
1. Work-family conflict	2.77	1.29	0.89					
2. Time pressure	3.53	1.21	0.51^∗∗∗^	0.87				
3. Problem solving	4.31	1.11	0.40^∗∗∗^	0.59^∗∗∗^	0.85			
4. Participation in decision making	3.96	1.04	-0.25^∗∗^	-0.38^∗∗∗^	-0.29^∗∗^	0.63		
5. Job autonomy	4.34	1.16	-0.34^∗∗∗^	-0.24^∗∗^	-0.01	0.31^∗∗∗^	0.92	
6. Support from supervisor	4.34	1.10	-0.36^∗∗∗^	-0.42^∗∗∗^	-0.24^∗∗^	0.68^∗∗∗^	0.36^∗∗∗^	0.86


*Cronbach’s alpha values are displayed along the diagonal of the correlation matrix.*

*^∗∗^*p* < 0.01. ^∗∗∗^*p* < 0.001.*

The results of the regression analyses are presented in Table 2 (M1–M3) and Table 3 (M4–M6). Overall, problem solving (M1–M3) and time pressure (M4–M6) were positively associated with WFC, and therefore H1a and H1b are supported. The interaction between problem solving and SS (M1) accounted for an additional 5% of the variance in WFC, *F*_change_(1, 118) = 8.13, *p* < 0.01. The same also occurred for the interaction between problem solving and PDM (M3), which accounted for an additional 3.8% of the variance in WFC, *F*_change_(1, 118) = 5.64, *p* < 0.05, whereas the interaction between problem solving and JA (M2) was not significant.

**Table 2:** Results from Moderated Multiple Regression Analyses: Model 1, Model 2, and Model 3 (*N* = 122).

	Model 1		Model 2		Model 3	
			
	*B*	*SE*	*B*	*SE*	*B*	*SE*
Problem solving	0.412^∗∗∗^	0.094	0.458^∗∗∗^	0.091	0.415^∗∗∗^	0.099
Support from supervisor	-0.249^∗^	0.098				
Job autonomy			-0.356^∗∗∗^	0.088		
Participation in decision making					-0.176	0.106
Problem solving × support	-0.204^∗∗^	0.072				
Problem solving × autonomy			-0.069	0.076		
Problem solving × participation					-0.185^∗^	0.078
Total *R*^2^	0.280		0.274		0.214	
Change in *R*^2^	0.050		0.005		0.038	


*All variables were centered at their means.*

*^∗^*p* < 0.05. ^∗∗^*p* < 0.01. ^∗∗∗^*p* < 0.001.*

**Table 3:** Results from Moderated Multiple Regression Analyses: Model 4, Model 5, and Model 6 (*N* = 122).

	Model 4		Model 5		Model 6	
			
	*B*	*SE*	*B*	*SE*	*B*	*SE*
Time pressure	0.476^∗∗∗^	0.090	0.473^∗∗∗^	0.084	0.499^∗∗∗^	0.090
Support from supervisor	-0.086	0.111				
Job autonomy			-0.246^∗∗^	0.087		
Participation in decision making					-0.041	0.106
Time pressure × support	-0.145^∗^	0.066				
Time pressure × autonomy			-0.078	0.061		
Time pressure × participation					-0.144^∗^	0.072
Total *R*^2^	0.316		0.324		0.292	
Change in *R*^2^	0.028		0.01		0.024	


*All variables were centered at their means.*

*^∗^*p* < 0.05. ^∗∗^*p* < 0.01. ^∗∗∗^*p* < 0.001.*

**FIGURE 2:**
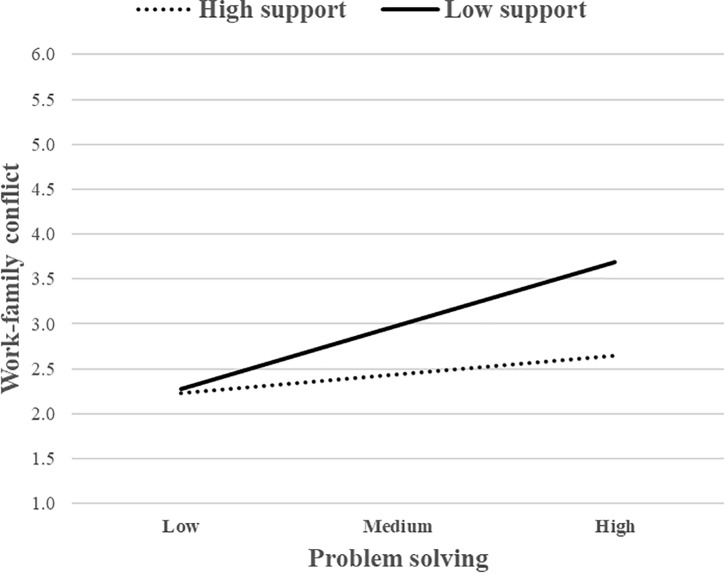
The moderating role of support from supervisor (SS) (job resource) in the relationship between problem solving (job demand) and WFC.

**FIGURE 3:**
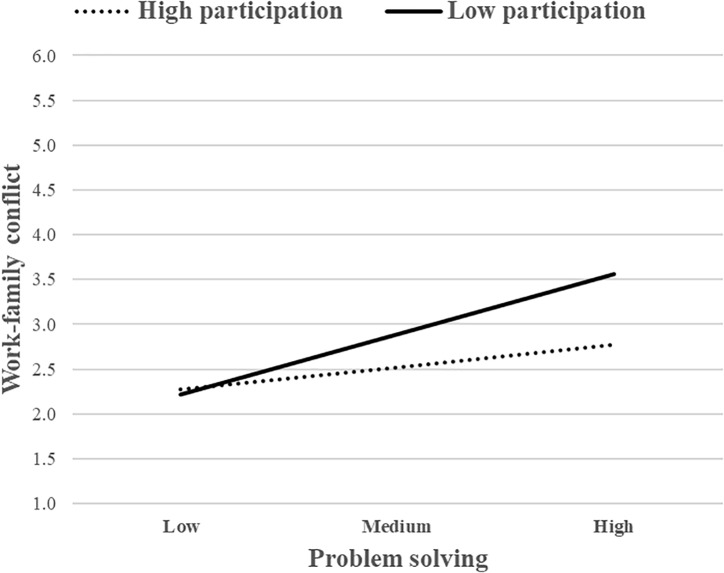
The moderating role of participation in decision making (PDM) (job resource) in the relationship between problem solving (job demand) and WFC.

To interpret the nature of the moderating effect, these interactions were presented graphically. The association between problem solving and WFC was stronger for individuals with low levels of SS (Figure 2). The same pattern also occurred for PDM (Figure 3). The simple slope analysis showed that the relationship between problem solving and WFC was positive and significant when SS was low (*b* = 0.64, *p* < 0.001) or when PDM was low (*b* = 0.61, *p* < 0.001), but non-significant when SS or PDM were high. These two job resources, therefore, buffered the positive association between problem solving and WFC, whereas JA did not. Overall, H2a was partially supported.

**FIGURE 4:**
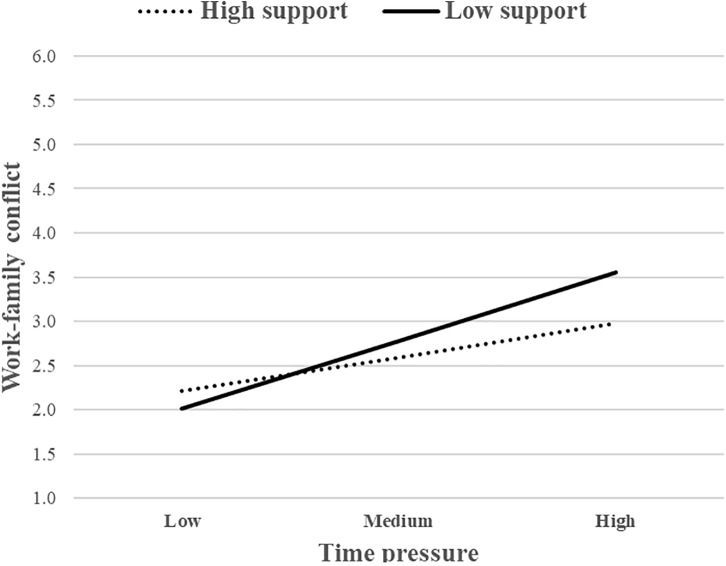
The moderating role of SS (job resource) in the relationship between time pressure (job demand) and WFC.

**FIGURE 5:**
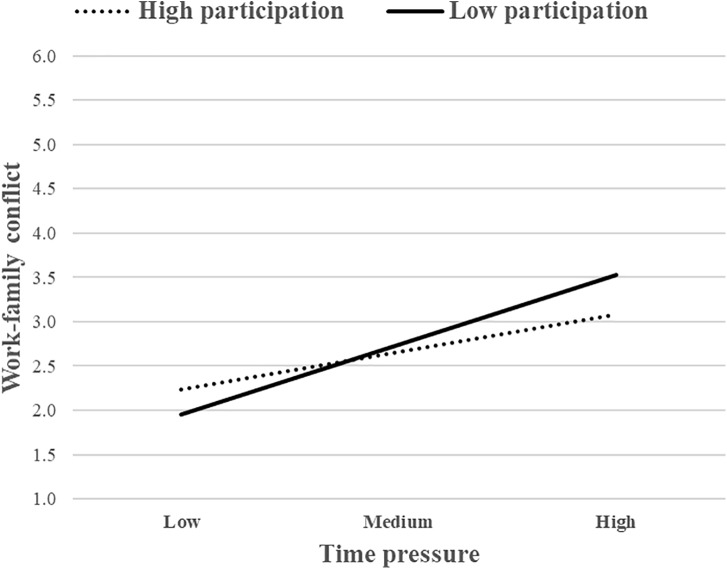
The moderating role of PDM (job resource) in the relationship between time pressure (job demand) and WFC.

Similarly, the interaction between time pressure and SS (M4) accounted for an additional 2.8% of the variance in WFC, *F*_change_(1, 118) = 4.86, *p* < 0.05, and the same also occurred for the interaction between time pressure and PDM (M6), which accounted for an additional 2.4% of the variance in WFC, *F*_change_(1, 118) = 4.01, *p* < 0.05. Again, the interaction between time pressure and JA (M5) was not significant. To interpret the nature of the moderating effect, these interactions were presented graphically. The association between time pressure and WFC was stronger for individuals with low levels of SS (Figure 4). The same pattern also occurred for PDM (Figure 5). The simple slope analysis showed that the relationship between time pressure and WFC was positive and significant either when SS was high (*b* = 0.32, *p* < 0.01) or low (*b* = 0.64, *p* < 0.001). The same pattern of results also occurred in M6, given that the relationship between time pressure and WFC was positive and significant either when PDM was high (*b* = 0.35, *p* < 0.01) or low (*b* = 0.65, *p* < 0.001). Overall, these two job resources buffered the positive association between time pressure and WFC, whereas JA did not. Therefore, H2b was partially supported.

## Discussion

Over the past decades, teachers have been increasingly exposed to external expectations and pressures (e.g., from supervisors, parents, and policy makers). As a consequence, teachers may experience higher workload (both teaching- and non-teaching-related), less time for interactions with colleagues, more conflict between work and family domains, and job burnout (Hargreaves, 1992; Ballet and Kelchtermans, 2009; Van Droogenbroeck et al., 2014). With the aim of preventing WFC and its consequences, the present study examined contextual work-related factors that may influence WFC among teachers in an Italian secondary school.

Our hypotheses were based on the JD-R model (Demerouti et al., 2001; Bakker and Demerouti, 2007, 2017), a relevant model of job stress and well-being that has recently been applied to the interface between work and private life (Bakker et al., 2011). Building on the JD-R, our first hypothesis was that job demands (i.e., risk factors) are positively associated with WFC, whereas our second hypothesis stated that job resources (i.e., protective factors) affect this association, which is expected to be particularly strong when JR are low. The results of this study largely support our predictions. First, job demands, in terms of qualitative and quantitative workload, were positively associated with WFC. Secondly, job resources, including SS and PDM, buffered this association, which was stronger when resources were low.

Overall, we believe that this study provides a valuable contribution to the literature on the interface between work and private life among teachers. First, our results suggest that workload, in terms of both the amount of work to be done in a given time (i.e., quantitative workload) and the difficulty or complexity of the job (i.e., qualitative workload), may play a central role in the onset of WFC in teachers. From a theoretical standpoint, this is consistent with the idea that demands at work deplete teachers’ personal resources (e.g., time, energy, and mood), which may lead to poorer outcomes in the family domain (e.g., reduced quality of care for family members), and WFC (Bakker et al., 2011; ten Brummelhuis and Bakker, 2012). Moreover, empirical research has shown workload to be positively associated with WFC (Grzywacz and Marks, 2000; Byron, 2005; Michel et al., 2011). Notably, Ilies et al. (2015) recently found in a sample of teachers that daily workload was associated with experiences of strain-based work–family conflict, and this relationship was mostly mediated by emotional fatigue. The authors argued that the teaching profession may drain individuals of the emotional resources that are needed at home to effectively take part in family life, thus resulting in strain-based WFC.

Secondly, our study showed that job resources, namely SS and PDM, may protect teachers against the negative consequences of workload, in terms of conflict between work and family domains. Accordingly, high workload may not necessarily result in WFC if teachers have adequate levels of job resources, which may help individuals to preserve or replenish personal resources such as time, energy, and mood (Bakker et al., 2011; ten Brummelhuis and Bakker, 2012). On a theoretical level, this is consistent with the buffer hypothesis of the JD-R, which claims that high job resources may offset the harmful impact of job demands on WFC (Bakker et al., 2011), job burnout (Bakker and Demerouti, 2007, 2017; Xanthopoulou et al., 2007), and work-related stress (Falco et al., 2018). Moreover, from an empirical standpoint, our findings are also consistent with the ones described by Bakker et al. (2011), who found that job resources, such as PDM and supervisory coaching, attenuated the adverse effect of job demands (i.e., work overload, emotional, and cognitive demands) on WFC among medical residents.

Interestingly, in our study JA did not buffer the association between workload and WFC. This is an intriguing finding, albeit not completely unexpected. Indeed, although JA plays a central role in models of work-related stress and motivation such as the job demand-control model (Karasek, 1979) and the job characteristics model (Hackman and Oldham, 1976), previous research has shown that JA does not attenuate the relationship between job demands (including workload) and WFC (Bakker et al., 2011; Falco et al., 2013a). A possible explanation is that, at least in certain circumstances (e.g., when confronted with tasks that can be performed at home, such as preparing for lessons and grading assignments), JA may contribute to blur the boundaries between work and private life (Blair-Loy, 2009). Another possible explanation is that teachers in our sample particularly benefit from other resources, such as SS and PDM, whenever confronted with their specific job demands, whereas the independence in scheduling the work and in determining the procedures to be used is less relevant to them. It is also possible that, compared to other job resources, teachers attribute less value to JA when coping with high demands at work, because autonomy is perceived as an intrinsic feature of their job. However, given the relative scarcity of studies on the possible moderating role of JA in the relationship between job demands and WFC among teachers, more research is warranted to generalize our findings.

Taken together, the findings of this study allow us to extend to the teaching profession the results of the seminal work by Bakker and colleagues, who claimed that “the JD-R model provides a fruitful framework for explaining which particular job designs facilitate, or instead prevent, work–home interference” (Bakker et al., 2011, p. 178). According to the JD-R, the specific combination of job demands and job resources should be considered, to understand which particular job designs may result in work–family conflict. In this regard, our study suggests that teachers with high levels of job resources, such as SS and PDM, can effectively cope with job demands, in terms of both qualitative and quantitative workload, thus preventing negative consequences such as WFC.

At a more general level, the results from this study are also in line with the Conservation of Resources (COR; Hobfoll, 1989, 2001) theory, which provides a theoretical framework to understand stress (acute and chronic) in various settings of people’s life. The COR theory claims that individuals try to acquire, retain and protect resources, which may include conditions (e.g., tenure, marriage, or occupational status), personal characteristics (e.g., self-esteem), or energies (e.g., time, money; Grandey and Cropanzano, 1999; Geurts and Demerouti, 2003). Psychological stress occurs when individuals’ resources are threatened with loss or actually lost, or when individuals do not gain adequate resources following a relevant investment of resources (Hobfoll, 1989, 2001). According to the COR theory perspective, the balancing of work and family roles may lead to a loss of resources and work family conflict (Grandey and Cropanzano, 1999; Richardson and Thompson, 2012). Consistently, our study showed that job demands, in terms of quantitative and qualitative workload, are associated with WFC in teachers.

Moreover, the COR theory claims that individuals have to invest resources to protect against resource loss (Hobfoll, 2001). With respect to the interface between work and home, this means that contextual demands in a domain (e.g., high demands at work) may result in an initial loss of resources (e.g., energy or time) with more immediate consequences in the other sphere (e.g., spousal conflict). To effectively cope with this situation and prevent further resource loss, individuals may invest additional resources, which might lead to a loss spiral over time (ten Brummelhuis and Bakker, 2012; Neto et al., 2016). In this perspective, individuals with greater resources are less vulnerable to resource loss than those with fewer resources (Hobfoll, 2001). With greater job resources available, conflict between work and family domains becomes less likely (Grandey and Cropanzano, 1999). Consistently, our study showed that greater resources at work may protect against the negative consequences of high job demands, in terms of WFC.

Interestingly, aging could play a role in this process. For example, teachers at the beginning of their career may experience higher levels of WFC, since they invest considerable amount of energy and personal resources to meet work goals and to deal with stressful work-related demands while, at the same time, they are also involved in parental duties (Antoniou et al., 2006). Conversely, older teachers may have lower levels of WFC, given that age is associated with greater job resources (e.g., tenure, status), coping resources, occupational experience, and organizational identification (Grandey and Cropanzano, 1999; Avanzi et al., 2012; Mauno et al., 2013). Future studies should investigate the possible moderating role of age in the relationship between job demands and resources on the one hand, and WFC and enrichment on the other.

Our study has some limitations. First, in this study we focused only on WFC, although it is possible that work and family domains may positively influence each other. For example, when resources are adequate to deal with high demands in one domain (e.g., at work), individuals may feel stimulated to learn and grow (e.g., in their job), and energies will be mobilized, rather than depleted (Geurts and Demerouti, 2003). This may in turn improve the quality of life in the other domain, such as family life (i.e., work-to-family enrichment; Greenhaus and Powell, 2006). Moreover, it should be noted that the cross-sectional design of the study precludes conclusions about the causal direction of the observed relationships. Future studies should investigate the longitudinal relationships between job demands, resources, and work family conflict, given that past research suggested that these constructs may influence each other over time (Demerouti et al., 2004). Moreover, studies using within person designs could shed light on the day-to-day processes through which work influences the family domain (Ilies et al., 2015). It should be also mentioned that the constructs of interests were determined using the same measurement method (i.e., self-report questionnaires), and the observed relationships could be affected by common method bias (Podsakoff et al., 2012). Accordingly, future studies could adopt, for example, observer-rating of WFC (e.g., spouse rating; Grandey et al., 2005), as well as biomarkers of stress that could be related to insufficient opportunities for recovery (Girardi et al., 2015a). Finally, future research should consider the possible role of additional job demands (e.g., emotional demands) and job resources (e.g., job security, support from colleagues, opportunities for professional development) that may be relevant for teachers (Xanthopoulou et al., 2007; Falco et al., 2008; Barbieri et al., 2014).

We believe that this study has relevant practical implications. More specifically, our results suggest that interventions should be aimed at preventing WFC, in order to avoid negative consequences for teachers and their students, such as job burnout, sickness absenteeism, and presenteeism, as well as reduced teachers’ effectiveness (Amstad et al., 2011; Falco et al., 2013b; Shen et al., 2015). These interventions should target the organization as well as the individuals (i.e., primary and secondary prevention).

Regarding primary prevention, organizations should be encouraged to optimize the balance between job demands and job resources (following a top-down approach; van Wingerden et al., 2017). On the one hand, this can be achieved by reducing job demands whenever possible, especially the ones perceived as hindrances (e.g., interpersonal conflict, role ambiguity, which may result in work overload), or minimize their negative consequences. For example, with respect to the time-based dimension of WFC, work activities that can interfere with teachers’ private lives, such as working overtime or working at home during the week-end, could be discouraged, whereas workers who reach their objectives operating within regular working hours could be rewarded. At the same time, to reduce strain-based WFC, activities that favor recovery should be promoted (Geurts and Sonnentag, 2006). For instance, teachers could learn simple physical exercises (e.g., stretching) or relaxation techniques to practice at home or at work, in specifically designated areas during work breaks (Taylor, 2005).

On the other hand, organizations should provide teachers with adequate levels of job resources, such as SS and PDM, in order to offset the negative consequences of job demands on WFC. Clearly, school leaders such as principals and vice principals play a central role in this process. For example, supervisors training programs could be directed at increasing the adoption of family supportive supervisor behaviors (Hammer et al., 2011). These behaviors refer to emotional support (e.g., listening and care for teachers’ work-family demands), instrumental support (e.g., providing effective support for day-by day teachers’ work-family demands), role-modeling behaviors (e.g., actively showing how to balance work and family domains), and creative work-family management (e.g., restructuring job to encourage teachers’ effectiveness in work and family domains). Moreover, school leaders could involve teachers in decision making (e.g., when new protocols are adopted or when resources are allocated), so that they can optimize their work processes. Furthermore, interventions could include case studies that adopt a situational approach for improving managerial competence in school leaders and teachers (Ripamonti and Scaratti, 2012; Scaratti and Ivaldi, 2015). In this respect, the active participation of different subjects (e.g., principals and vice principals, teachers, clerical workers), including practitioners such as work and organizational psychologists or therapists, is essential to gain a deeper insight into the specific issues reported and develop effective organizational policies and practices (Cunliffe and Scaratti, 2017).

With respect to secondary prevention, interventions should be directed at the identification and training of the workers at risk of WFC. Again, school leaders play a key role because they are able to identify teachers who may need support in balancing work and family domains, as well as benefit from specific interventions. In this regard, specific training programs could help teachers to develop personal resources, such as self-esteem, self-efficacy, and courage (De Stasio et al., 2017; Farnese et al., 2017; Magnano et al., 2017; Benevene et al., 2018b,c) as well as skills needed to effectively cope with high workload and time pressure (e.g., time management, goal-setting; Richardson and Rothstein, 2008). This in turn may also improve students’ personal resources, motivation, satisfaction, and performance (Magnano et al., 2014a,b; Ripamonti et al., 2018; Santisi et al., 2018). The aforementioned interventions should be directed specifically at individuals with high levels of personal demands (e.g., perfectionism; Falco et al., 2014), which may influence the effort they invest in their work (Bakker and Demerouti, 2017). For example, individuals with high levels of perfectionism could be trained to gain awareness about the dysfunctional role played by work-related irrational beliefs (Girardi et al., 2015b; Falco et al., 2017). Finally, interventions should be aimed at fostering job crafting, in which teachers are encouraged to proactively optimize their own job designs (bottom-up approach; Tims et al., 2012). For example, teachers could try to increase social resources (Benevene et al., 2018a), such as seeking social support from the school leader when confronted with high demands at work, or more structural resources, such as a more active PDM.

Overall, building on the seminal study conducted by Bakker et al. (2011) on a sample of medical residents, the aim of this paper was to extend the Job Demands–Resources model to WFC in teachers. Our results showed that job demands, in terms of qualitative and quantitative workload (i.e., problem solving and time pressure, respectively), were positively associated with WFC in teachers. Furthermore, in line with the buffer hypothesis of the JD-R, job resources, in terms of SS and PDM, modulated the association between job demands and WFC, which was stronger when resources were low. Therefore, in order to prevent WFC among teachers, job designs that optimize the balance between job demands and resources should be encouraged.

## Ethics Statement

All participants gave their written informed consent before the administration of the questionnaire, in accordance with the Declaration of Helsinki. The study was carried out in accordance the rules of AIP (Associazione Italiana di Psicologia – Italian Association of Psychology), according to which there was no need for previous ethics approval, since it would not deal with animals or vulnerable groups, or would involve risk for the well-being of participants, or use biomedical devices, or invasive investigation tools. Our study did not need ethics approval, according to our national regulations as well as to the Ethics Committee of the University of Padova.

## Author Contributions

ADC developed the research project, with the contribution of DG, AF, and LDC. DG carried out the data analysis, with the contribution of ADS. ADC reviewed the literature. AF and LDC developed the questionnaire. ADS developed the administration procedure and prepared the data set.

## Conflict of Interest Statement

The authors declare that the research was conducted in the absence of any commercial or financial relationships that could be construed as a potential conflict of interest.
